# mRNA Expression and Activity of Nucleoside Transporters in Human Hepatoma HepaRG Cells

**DOI:** 10.3390/pharmaceutics10040246

**Published:** 2018-11-21

**Authors:** Abdullah Mayati, Amélie Moreau, Elodie Jouan, Marie Febvre-James, Claire Denizot, Yannick Parmentier, Olivier Fardel

**Affiliations:** 1 Univ Rennes, Inserm, EHESP, IRSET (Institut de Recherche en Santé, Environnement et Travail) - UMR_S 1085, F-35000 Rennes, France; mayati1980@hotmail.com (A.M.); elodie.jouan@gmail.com (E.J.); marie.febvre-james@univ-rennes1.fr (M.F.-J.); 2 Centre de Pharmacocinétique, Technologie Servier, F-45000 Orléans, France; amelie.moreau@servier.com (A.M.); claire.denizot@servier.com (C.D.); yannick.parmentier@servier.com (Y.P.); 3 Pôle Biologie, Centre Hospitalier Universitaire, F-35033 Rennes, France

**Keywords:** ENT1, nucleoside transporter, HepaRG cells, hepatocytes, uridine

## Abstract

The HepaRG cell line is a highly differentiated human hepatoma cell line, displaying the expression of various drug transporters. However, functional expression of nucleoside transporters remains poorly characterized in HepaRG cells, although these transporters play a key role in hepatic uptake of antiviral and anticancer drugs. The present study was, therefore, designed to characterize the expression, activity and regulation of equilibrative (ENT) and concentrative (CNT) nucleoside transporter isoforms in differentiated HepaRG cells. These cells were found to exhibit a profile of nucleoside transporter mRNAs similar to that found in human hepatocytes, i.e., notable expression of ENT1, ENT2 and CNT1, with very low or no expression of CNT2 and CNT3. ENT1 activity was, next, demonstrated to be the main uridine transport activity present in HepaRG cells, like in cultured human hepatocytes. Various physiological factors, such as protein kinase C (PKC) activation or treatment by inflammatory cytokines or hepatocyte growth factor (HGF), were additionally found to regulate expression of ENT1, ENT2 and CNT1; PKC activation and HGF notably concomitantly induced mRNA expression and activity of ENT1 in HepaRG cells. Overall, these data suggest that HepaRG cells may be useful for analyzing cellular pharmacokinetics of nucleoside-like drugs in human hepatic cells, especially of those handled by ENT1.

## 1. Introduction

The hepatic cell line HepaRG is an original human hepatoma cell line, which expresses various liver-specific functions when cultured in appropriate conditions, i.e., in the presence of 2% (vol/vol) dimethyl sulfoxide (DMSO) [1]. These hepatoma cells have, consequently, been proposed as surrogates for the use of primary human hepatocytes, especially for xenobiotic metabolism and toxicity studies [2,3]. This assertion is notably validated by the fact that HepaRG cells display functional activities of main drug hepatic detoxifying proteins, including drug metabolizing enzymes like cytochromes P-450 [4], but also drug transporters [5], whose major role in hepatobiliary elimination of xenobiotics is now well-established. Importantly, HepaRG cells exhibit polarized expression of drug transporters, with expression of solute carrier (SLC) transporters like organic cation transporter 1 (OCT1/*SLC22A1*) and organic anion transporting polypeptides (OATPs/*SLCO*s) 1B1 (OATP1B1/*SLCO1B1*) and 2B1 (OATP2B1/*SLCO2B1*) at their sinusoidal pole, and expression of drug efflux ATP-binding cassette (ABC) transporters like P-glycoprotein (*ABCB1*) and multidrug resistance-associated protein 2 (MRP2/*ABCC2*) at their canalicular pole [6]. Expression of drug transporters in HepaRG cells thus mimicks that displayed by primary human hepatocytes [7].

Concentrative and equilibrative nucleoside transporters (CNT/*SLC28* and ENT/*SLC29*) are two families of transmembrane proteins that mediate transport of hydrophilic nucleosides across cell membranes [8,9]. The CNT family includes three members, i.e., CNT1 (*SLC28A1*), CNT2 (*SLC28A2*) and CNT3 (*SLC28A3*), which are sodium-dependent symporters, that mediate unidirectional transport of nucleosides into cells [10]. The ENT family comprises four members, i.e., ENT1 (*SLC29A1*), ENT2 (*SLC29A2*), ENT3 (*SLC29A3*) and ENT4 (*SLC29A4*) [11]. ENT1 and ENT2 mediate passive bidirectional transport of purine and pyrimidine nucleosides down a concentration gradient across the plasma membrane; ENT3 transports nucleosides across intracellular membranes, notably those of lysosomes [12], whereas ENT4, also known as plasma membrane monoamine transporter (PMAT), mainly functions as a polyspecific organic cation transporter [13], handling only adenosine among nucleosides [14]. Thereby, only ENT1 and ENT2 among ENTs can be considered as prototypical plasma membrane nucleoside transporters. Unlike the broad specificity of ENT1/2 and CNT3, CNT1 and CNT2 primarily handle pyrimidine and purine nucleosides, respectively [15]. Uridine is an ubiquitous substrate for CNTs and ENT1/2 [16]. Both CNTs and ENTs are likely to contribute to the cellular pharmacokinetics and, in this way, to the pharmacological activity of various nucleoside analogs used in antiviral or anticancer therapy [17]. 

Nucleoside transporters are expressed in hepatocytes, notably ENT1, ENT2 and CNT1 [18,19]. They have been implicated, especially ENT1, in the hepatic uptake of the antiviral agent ribavirin [20,21], as well as in that of the anticancer drug gemcitabine [22]. However, whether HepaRG cells exhibit functional expression and regulation of nucleoside transporters remains unknown. Nevertheless, this point is worthy of interest, owing to (i) the established role played by nucleoside transporters in pharmacokinetics and toxicity of various drugs and xenobiotics; and (ii) the growing use of HepaRG cells as surrogates for human hepatocytes in pharmacological and toxicological studies [2,3]. Therefore, the present study was designed to characterize the expression of plasma membrane nucleoside transporters, i.e., ENT1, ENT2, CNT1, CNT2 and CNT3, in HepaRG cells, as well as the putative regulation of some of them by factors known to modulate drug transporter expression in human hepatic cells, like protein kinase C (PKC) activation [23] or inflammatory cytokine treatment [24]. 

## 2. Materials and Methods 

### 2.1. Chemicals and Reagents

*S*-(4-Nitrobenzyl)-6-thioinosine (NBMPR), phorbol myristate acetate (PMA), lipopolysaccharide (LPS) (*Escherichia coli* O55:B5) and bovine insulin were provided by Sigma-Aldrich (Saint-Quentin Fallavier, France). Ruxolitinib was from Selleckchem (Houston, TX, USA), whereas the PKC inhibitors GF 109203X and Gö 6976 were from Calbiochem (La Jolla, CA, USA). Recombinant human hepatocyte growth factor (HGF), tumor necrosis factor (TNF) α, interleukin (IL) 6 and IL1β were purchased from R&D Systems (Minneapolis, MN, USA). [5-^3^H]-uridine (specific activity = 20.4 Ci/mmol) was from PerkinElmer (Courtaboeuf, France). All other reagents were commercial products of the highest purity available.

### 2.2. Cell Culture

HepaRG cells were routinely plated at low density (27,000 cells/cm^2^) and cultured in Williams’ E medium supplemented with 10% (vol/vol) fetal calf serum, 20 µg/mL streptomycin, 20 IU/mL penicillin, 2 mM glutamine, 5 µg/mL bovine insulin, and 50 µM hydrocortisone hemisuccinate. After two weeks, cells were trypsinated for passaging or cultured for additional two weeks in the same medium added with 2% (vol/vol) DMSO, in order to obtain differentiated hepatocytes-like cells, as previously described [1]. 

Freshly isolated human hepatocytes were obtained from the Biological Resource Center BB-0033-00056 (University Hospital, Rennes, France), which has obtained the authorization No DC-2008-630 from the French Ministry of Health to collect hepatic resections from the digestive surgery department and then to isolate and deliver the hepatocytes. All liver fragment donors were adult and provided a written informed consent to participate in the study. All experimental procedures complied with French laws and regulations; they were approved by the National Ethics Committee from INSERM (IRB00003888). Upon delivery, human hepatocytes were seeded on plastic dishes at a high density (250,000 cells/cm^2^) in Williams’ E medium, supplemented with 10% (vol/vol) fetal calf serum, 5 μg/mL bovine insulin, 20 IU/mL penicillin, 20 μg/mL streptomycin, and 2 mM glutamine. After a 24 h-seeding culture period, the medium was discarded and primary hepatocytes were next cultured for 6 days in the DMSO-containing HepaRG cell-differentiating medium described above, as previously described [7]. 

The human hepatoma cell line HuH-7 was cultured in Dulbecco’s modified Eagle medium (DMEM, Life Technologies), supplemented with 10% (vol/vol) fetal calf serum, 20 IU/mL penicillin and 20 μg/mL streptomycin, as previously described [25].

Human macrophages were obtained from peripheral blood monocytes as previously reported [26]. Briefly, peripheral blood mononuclear cells were first isolated from blood buffy coats of healthy donors through Ficoll gradient centrifugation. After a 1 h adhesion step, the cells were cultured for 6 days in RPMI 1640 medium, supplemented with 10% (vol/vol) fetal calf serum, 2 mM glutamine, 20 IU/mL penicillin and 20 μg/mL streptomycin, in the presence of 400 IU/mL GM-CSF.

Nearly-haploid HAP1 cells and ENT1-knockout HAP1 (HAP1 ENT1^△^) cells, edited by CRISPR/Cas9 to contain a 14 bp deletion in a coding exon of ENT1, were obtained from Horizon Discovery (Cambridge, UK). They were routinely cultured in Iscove’s modified Dulbecco’s medium (IMDM) (Thermo Fisher Scientific) supplemented with 10% (vol/vol) fetal calf serum, 20 IU/mL penicillin and 20 μg/mL streptomycin.

### 2.3. RNA Isolation and Analysis 

Total RNAs were extracted from cells using the TRI reagent (Sigma-Aldrich), and were then reverse-transcribed to cDNA using the reverse-transcription (RT) kit from Applied Biosystems (Foster City, CA, USA). Quantitative polymerase chain reaction (qPCR) assays were next performed using the fluorescent dye SYBR Green methodology and a CFX384 real-time PCR detection system (Bio-Rad, Marnes-la-Coquette, France), as previously described [6]. Gene primers were: CNT1 sense, AGGTCCTGCCCATCATTGTC, CNT1 anti-sense, CAAGTAGGGCCGGATCAGTA, CNT2 sense, AATGGGTGTTTGCAGGAGTC, CNT2 anti-sense, GAAGACCTAGGCCCGAAAAC, CNT3 sense, GACTCACATCCATGGCTCCT, CNT3 antisense, TTCCAGGGAAAGTGGAGTTG, ENT1 sense, CCTGGCTTTCTCTGTCTGCT, ENT1 anti-sense, AGTAACGTTCCCAGGTGCTG, ENT2 sense, CCCTGGATCTTGACCTGGAG, ENT2 anti-sense, GGTTTTCCTGGCTTCTGGG, 18S rRNA sense, CGCCGCTAGAGGTGAAATTC and 18S rRNA anti-sense, TTGGCAAATGCTTTCGCT. The specificity of each gene amplification was verified at the end of qPCR reactions through analysis of dissociation curves of the PCR products. Amplification curves were analyzed with the CFX Manager software (Bio-Rad), using the comparative cycle threshold method. Relative quantification of the steady-state target mRNA levels was calculated after normalization of the total amount of cDNA tested to the 18S rRNA endogenous reference, using the 2^(−ΔΔCt)^ method. Even if the use of several reference genes has been preconized [27], that of the 18S rRNA as a unique reference gene has been previously successfully retained for investigating transporter mRNA regulation in response to PMA or cytokines in HepaRG cells [23,28]. Indeed, the amount of 18S rRNA is presumed to remain constant, whatever the cell culture conditions. In agreement with this assertion, the amount of 18S rRNA in proliferating and differentiated HepaG cells did not vary, i.e., the C_t_ numbers determined from qPCR assays were similar in these cells (Figure S1). Data of qPCR experiments were finally commonly expressed in arbitrary units relatively to 18S rRNA or as fold change comparatively to control untreated cells, as previously reported [29].

### 2.4. Immunofluorescence Assays

Cells were fixed with ice-cold acetone for 10 min, washed with phosphate-buffered saline (PBS) and incubated in a 4% (weight/vol) bovine serum albumin/PBS blocking solution for 1 h at room temperature. Cells were next incubated overnight with 1:50 (vol/vol) working dilutions (4 µg/mL) of primary monoclonal mouse antibodies targeting CNT1 (clone G-1), ENT1 (clone F-12) or ENT2 (clone A-8), provided by Santa Cruz Biotechnology (Dallas, TX, USA). Cells were then washed twice with PBS, before adding secondary antibody directed against mouse IgG and coupled to Alexa Fluor 488 (Cell Signaling, Leiden, The Netherlands), for 45 min. In some experiments, additional labelling of F-actin with Alexa Fluor 555-coupled phalloidin (Ozyme, Montigny-le-Bretonneux, France) was performed for 45 min. Nuclei were next stained by 4′,6-diamidino-2-phenylindole (DAPI) for 10 min. Cells were finally washed three times with PBS and images of immuno-labeling were acquired with a confocal fluorescence microscope LEICA DMI 6000 CS (Leica Microsystemes SAS, Nanterre, France).

### 2.5. Transport Assays

For transport assays, uridine was used as a specific and common substrate for CNT1, CNT2, CNT3, ENT1 and ENT2 [9]. Uridine is also a substrate for ENT3, but the intracellular location of this transporter discards any role in initial cellular uptake of uridine [12], whereas ENT4 fails to transport uridine [9]. To discriminate among CNT and ENT activities, we used the differential sensitivity of these transporters to sodium withdrawal and to inhibitors such as NBMPR (which inhibits ENT1 when used at 100 nM and ENT1 and ENT2 when used at 100 µM, but not CNTs) [30], thymidine (blocking CNT1 and CNT3) [19] and inosine (blocking CNT2 and CNT3) [31] (Table 1). 

Cells were incubated with 24.5 nM [^3^H]-uridine for 5 min at 37 °C, in a well-defined transport assay buffer consisting of 5.3 mM KCl, 1.1 mM KH_2_PO_4_, 0.8 mm MgSO_4_, 1.8 mM CaCl_2_, 11 mM d-glucose, 10 mM 4-(2-hydroxyethyl)-1-piperazineethanesulfonic acid (HEPES), and 136 mM *N*-methyl-glucamine (sodium-free buffer) or 136 mM NaCl (sodium-containing buffer) and adjusted to pH 7.4, in the presence or absence of NBMPR (used at 100 nM or 100 µM), 200 µM inosine or 200 µM thymidine. After washing in PBS, cells were lysed, and accumulation of radiolabeled uridine was determined through scintillation counting and normalized to total protein content, determined by the Bradford method. Nucleoside transporter activities related to uridine uptake were next determined using the following equations:

ENT_Total_ activity = U_-Na_ − U_-Na/+ 100 µM NBMPR_ (1)

ENT1 activity = U_-Na_ − U_-Na/+ 100 nM NBMPR_ (2)

ENT2 activity = ENT_Total_ activity − ENT1 activity (3)

CNT_Total_ activity = U_+Na/+ 100 µM NBMPR_ − U_-Na/+ 100 µM NBMPR_ (4)

CNT1 activity = U_+Na/+ 100 µM NBMPR/+ 200 µM Inosine_ − U_-Na/+ 100 µM NBMPR_ (5)

CNT2 activity = U_+Na/+ 100 µM NBMPR/+ 200 µM Thymidine_ − U_-Na/+ 100 µM NBMPR_ (6)

CNT3 activity = CNT_Total_ activity − (CNT1 activity + CNT2 activity) (7)

with U_-Na_ = uridine uptake in the absence of sodium, U_-Na/+ 100 µM NBMPR_ = uridine uptake in the absence of sodium and presence of 100 µM NBMPR (this corresponds to passive uptake of uridine, when CNTs and ENTs are not functional), U_+Na/+ 100 µM NBMPR_ = uridine uptake in the presence of sodium and 100 µM NBMPR, U_+Na/+ 100 µM NBMPR/+ 200 µM Inosine_ = uridine uptake in the presence of sodium, 100 µM NBMPR and 200 µM inosine and U_+Na/+ 100 µM NBMPR/+ 200 µM Thymidine_ = uridine uptake in the presence of sodium, 100 µM NBMPR and 200 µM thymidine.

### 2.6. Statistical Analysis

Quantitative data were routinely expressed as means ± standard error of the mean (S.E.M.) of at least three independent assays. Statistical analysis was performed using the Student’s *t* test or by analysis of variance (ANOVA) followed by Dunnett’s or Tukey’s post hoc tests. The criterion of significance was *p* < 0.05.

## 3. Results

### 3.1. Expression of Nucleoside Transporters in HepaRG cells

**Figure 1:**
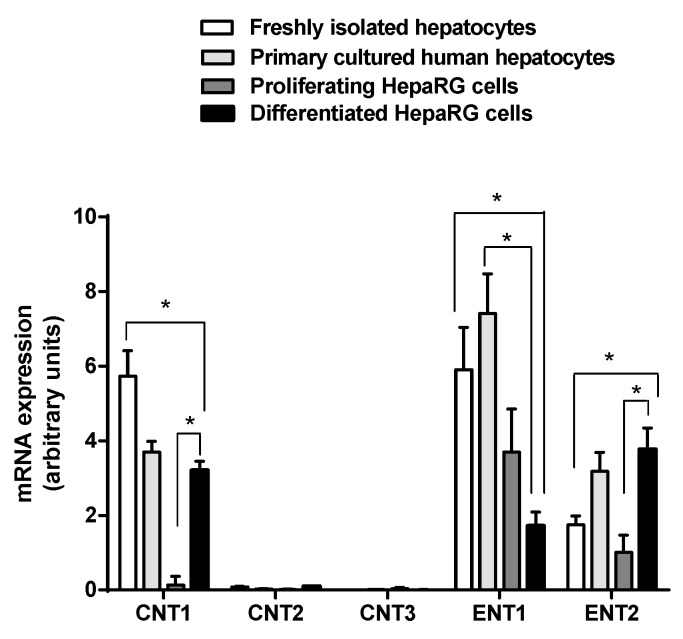
Expression of nucleoside transporter mRNAs in proliferating and differentiated HepaRG cells and in freshly isolated and primary cultured human hepatocytes. Expression of CNT1, CNT2, CNT3, ENT1 and ENT2 mRNAs was analyzed by reverse-transcription quantitative polymerase chain reaction (RT-qPCR). Data are expressed in arbitrary units and are the means ± S.E.M. of values from at least seven independent human hepatocyte populations and five independent HepaRG cell cultures. *, *p* < 0.05.

Expression of nucleoside transporter mRNAs was determined by RT-qPCR in proliferating HepaRG cells (cells cultured for 3 days after initial seeding, without DMSO) and in differentiated HepaRG cells (cells cultured for 15 days after seeding without DMSO, followed by an additional 15 day-culture period in the presence of DMSO), as well as in freshly isolated hepatocytes and primary cultured human hepatocytes. As indicated in Figure 1, ENT1 and ENT2 were expressed at substantial levels in freshly isolated and primary hepatocytes, and in proliferating and differentiated HepaRG cells. ENT1 mRNA expression in differentiated HepaRG cells was, however, significantly lower than those found in freshly isolated and cultured hepatocytes; it was also lower than that found in proliferating HepaRG cells, even if statistical significance was not reached. ENT2 mRNA expression was significantly higher in differentiated HepaRG cells than in freshly isolated hepatocytes and proliferating HepaRG cells (Figure 1). CNT1 mRNAs were expressed at substantial level in freshly isolated and primary cultured hepatocytes, as well as in differentiated HepaRG cells, but were barely detected in proliferating HepaRG cells. CNT1 mRNA level in differentiated HepaRG cells was similar to that found in primary human hepatocytes and slightly, but significantly, lower than that observed in freshly isolated human hepatocytes. Both CNT2 and CNT3 were not expressed, or only at a very low level, in hepatocytes and proliferating and differentiated HepaRG cells (Figure 1).

**Figure 2:**
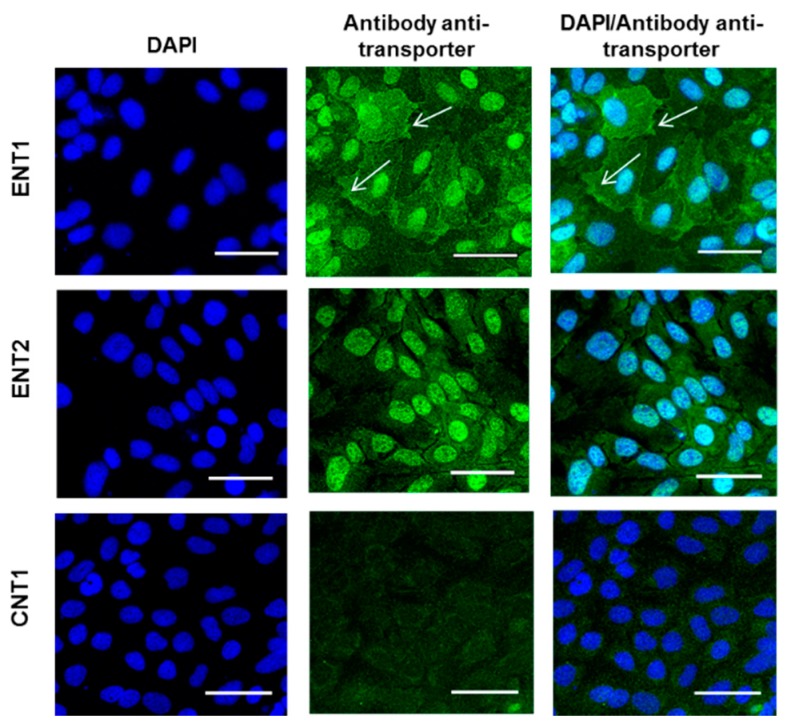
Immunolocalization of ENT1, ENT2 and CNT1 in proliferating HepaRG cells. ENT1, ENT2 and CNT1 were immunolocalized in proliferating HepaRG cells, as described in Materials and Methods. Nucleoside transporter immunolabeling appears as green fluorescence on microscopy pictures, whereas 4′,6-diamidino-2-phenylindole (DAPI)-stained nuclei are blue. White arrows indicate membrane labelling. Data are representative of three independent assays. White bar = 50 µm.

**Figure 3:**
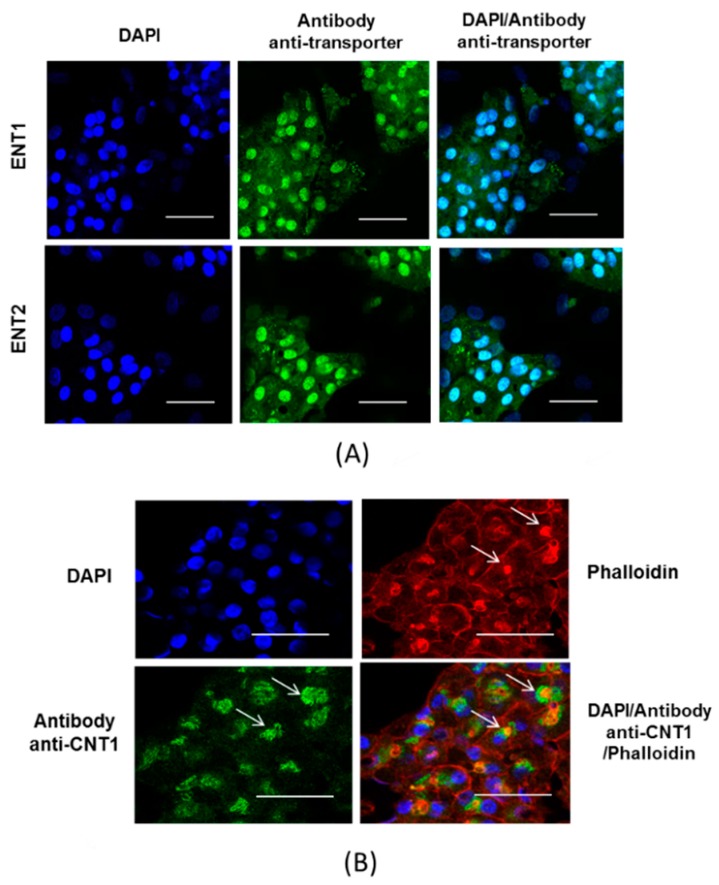
Immunolocalization of ENT1, ENT2 and CNT1 in differentiated HepaRG cells. (**A**) ENT1 and ENT2 were immunolocalized in differentiated HepaRG cells, as described in Materials and Methods. Nucleoside transporter immunolabeling appears as green fluorescence on microscopy pictures, whereas DAPI-stained nuclei are blue. (**B**) CNT1 and phalloidin-labeled F-actin were co-localized in differentiated HepaRG cells. White arrows indicate pericanalicular labeling of CNT1 and F-actin. (**A**,**B**) Data are representative of three independent assays. White bar = 50 µm.

Protein expression of CNT1, ENT1 and ENT2 was next studied by immunofluorescence in proliferating and differentiated HepaRG cells. As shown in Figure 2, ENT1 was detected at the plasma membrane and in the nuclei of proliferating cells, whereas ENT2 was mostly located in the nuclei. CNT1 was by contrast not detected in proliferating cells. Differentiated HepaRG cells mainly exhibited labeling of nuclei for both ENT1 and ENT2 (Figure 3A). Differentiated HepaRG cells also expressed CNT1, with an apparent CNT1-labeling localized close to that due to phalloidin (Figure 3B). Phalloidin labeling is known to highlight pericanalicular F-actin network, distributed around bile canaliculus-like structures [32]; therefore, this suggests a canalicular or pericanalicular location of CNT1 in differentiated HepaRG cells. 

### 3.2. Nucleoside Transporter Activities in HepaRG Cells

**Figure 4:**
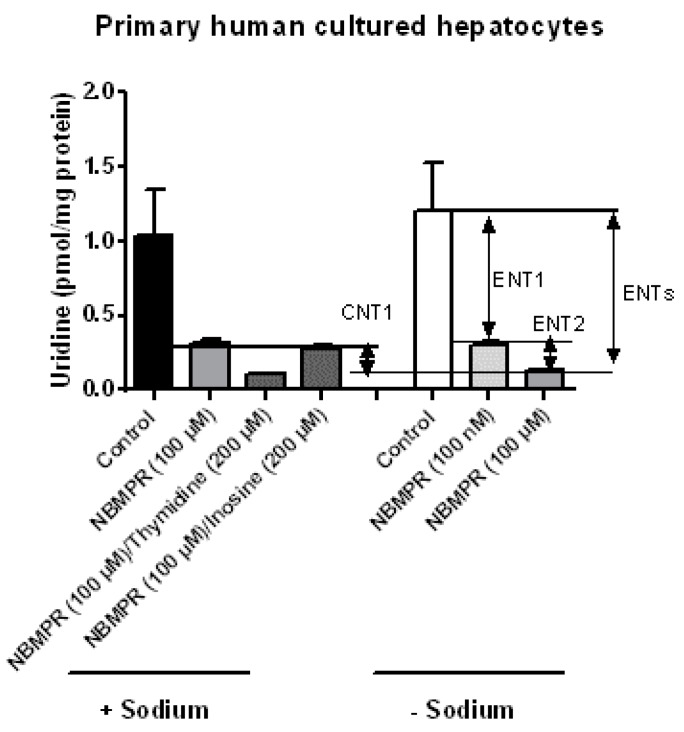
Uridine uptake in primary cultured human hepatocytes. Primary human hepatocytes were incubated with 24.5 nM [^3^H]-uridine for 5 min at 37 °C, in the presence or absence of sodium and in the presence or absence of the ENT inhibitor NBMPR (used at 100 nM or 100 µM) or the CNTs inhibitor thymidine or inosine. Uridine accumulation was next determined by scintillation counting. Data are expressed as pmol uridine/mg protein and are the means ± S.E.M. of values from four independent human hepatocyte populations. Activities of CNT1, ENTs, ENT1 and ENT2, defined by equations described in Materials and Methods, are indicated by double arrows on the graphs.

Analysis of nucleoside transporter activities was based on the measurement of uridine uptake in the absence or presence of sodium and/or various inhibitors. These methods highlighted CNT1, ENT1 and ENT2 activities in primary human hepatocytes, whereas CNT2 and CNT3 activities were not detected (Figure 4). Such a profile of nucleoside transporter activities fully agrees with that of nucleoside transporter mRNA expression, i.e., expression of CNT1, ENT1 and ENT2 mRNAs in primary human hepatocytes (Figure 1). However, it is noteworthy that total uridine uptake in human hepatocytes in the presence of sodium (both CNTs and ENTs are functional) is not higher than that in the absence of sodium (only ENTs are functional). This may be due to the fact that CNT1 activity is low when compared to ENT1 activity (Figure 4) and/or to the reduced number (four) of independent hepatocyte populations analyzed in the functional assay. Alternatively, ENT1 is an equilibrative bidirectional transporter [21] and it may be hypothesized that, when CNT1 activity is functional (in the presence of sodium), high uridine intracellular concentration may be reached, which may restrain ENT1 activity.

**Figure 5:**
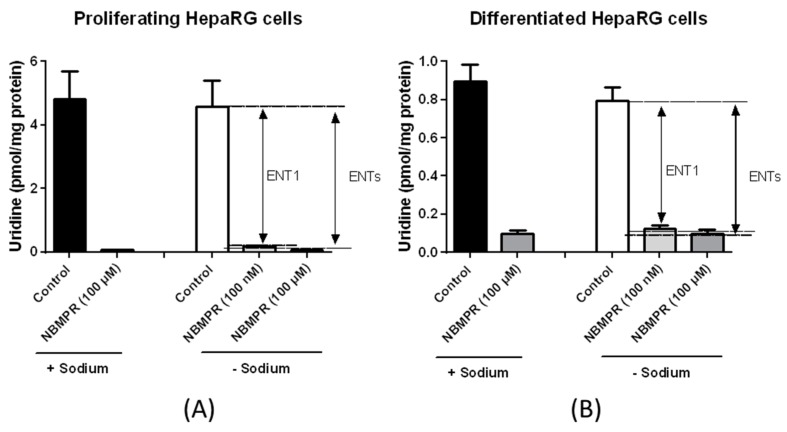
Uridine uptake in proliferating (**A**) and differentiated (**B**) HepaRG cells. Proliferating and differentiated HepaRG cells were incubated with 24.5 nM [^3^H]-uridine for 5 min at 37 °C, in the presence or absence of sodium and in the presence or absence of the ENT inhibitor NBMPR (used at 100 nM or 100 µM). Uridine accumulation was next determined by scintillation counting. Data are expressed as pmol uridine/mg protein and are the means ± S.E.M. of values from at least four independent HepaRG cell cultures. Activities of nucleoside transporters, defined by equations described in Materials and Methods, are indicated by double arrows on the graphs.

To further confirm the relevance of the uridine uptake-based assay for discriminating among nucleoside transporter activities, we performed it in various cell types differentially expressing nucleoside transporters. Hepatoma HuH-7 cells, which exhibited mainly ENT2 mRNA expression (Figure S2A, left panel), displayed marked ENT2 activity (Figure S2A, right panel). Primary human macrophages, which highly expressed CNT3 (Figure S2B, left panel)), concomitantly exhibited marked CNT3 activity (Figure S2B, right panel). Finally, HAP1 cells displayed membrane expression of ENT1 (Figure S3A) and ENT1 activity (Figure S3B); by contrast, HAP1 ENT1^△^ cells failed to exhibit membrane expression of ENT1 (Figure S3A) and ENT1 activity (Figure S3B). Taken together, these data support the conclusion that the uridine uptake-based assay permits to detect specific activity of nucleoside transporters when they are substantially expressed. Consequently, we applied this assay to proliferating and differentiated HepaRG cells. CNT total activity, i.e., the uptake of uridine in the presence of sodium and 100 µM NBMPR minus that in the absence of sodium and presence of 100 µM NBMPR, was not detected or only at a very low level in both proliferating and differentiated HepaRG cells (Figure 5); consequently, the effects of the CNT inhibitors thymidine and inosine were not analyzed. By contrast, ENT1 activity was detected in both proliferating and differentiated HepaRG cells and corresponded to nearly total ENT activity, indicating that ENT2 activity was barely detectable (Figure 5). 

**Figure 6:**
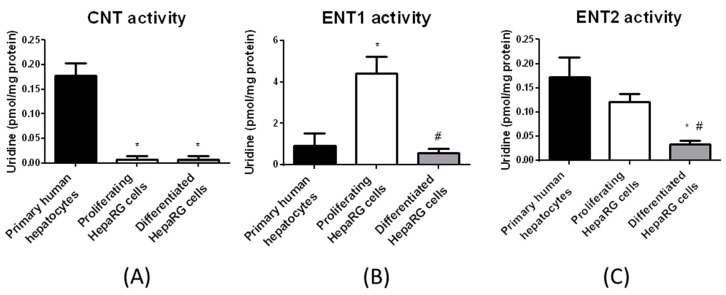
Comparison of CNT (**A**), ENT1 (**B**) and ENT2 (**C**) activities in primary cultured human hepatocytes and proliferating and differentiated HepaRG cells. CNT, ENT1 and ENT2 activities based on uridine uptake were calculated from values reported in Figure 5, using equations described in the Material and Methods. Data are expressed as pmol uridine/mg protein and are the means ± S.E.M. of values from four independent human hepatocyte populations and at least four independent HepaRG cell cultures. *, *p* < 0.05 when compared to primary cultured human hepatocytes; #, *p* < 0.05 when compared to proliferating HepaRG cells.

Activities of ENT1, ENT2 and CNTs in primary cultured human hepatocytes and HepaRG cells were next calculated using Equations (2)–(4), and compared with each other (Figure 6). Primary human hepatocytes exhibited the highest CNT activity, mainly related to CNT1 as indicated above, and also the highest ENT2 activity. Proliferating HepaRG cells displayed the highest ENT1 activity. Differentiated HepaRG cells exhibited an ENT1 activity similar to that of primary human hepatocytes, whereas they displayed lower ENT2 activity and barely detectable, if any, CNT activity (Figure 6). 

**Figure 7:**
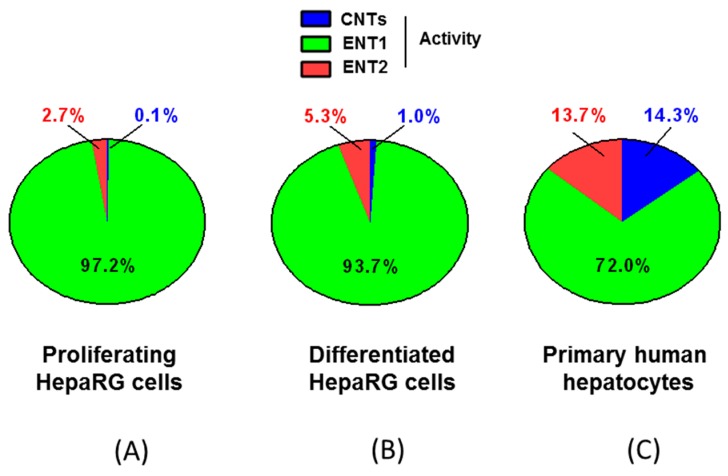
Graphical representation of the relative contribution of CNTs, ENT1 and ENT2 activities to total uridine uptake in proliferating (**A**) and differentiated (**B**) HepaRG cells and primary human hepatocytes (**C**). Percentages indicated on graphs correspond to the percentages of contribution.

When considering total nucleoside transporter activity, i.e., the sum of CNT, ENT1 and ENT2 activity-based uridine uptake, ENT1 activity accounted for more than 90% of uridine uptake for both proliferating and differentiated HepaRG cells (Figure 7). It was also the main contributor to uridine uptake in primary human hepatocytes, but in a less marked manner (it represented 72% of total uridine uptake). The relative contribution of CNT and ENT2 activities to uridine uptake were substantial (around 14% for each of them) only in primary cultured human hepatocytes (Figure 7).

### 3.3. Regulation of Nucleoside Transporter Expression by Protein Kinase C (PKC) Activation

**Figure 8:**
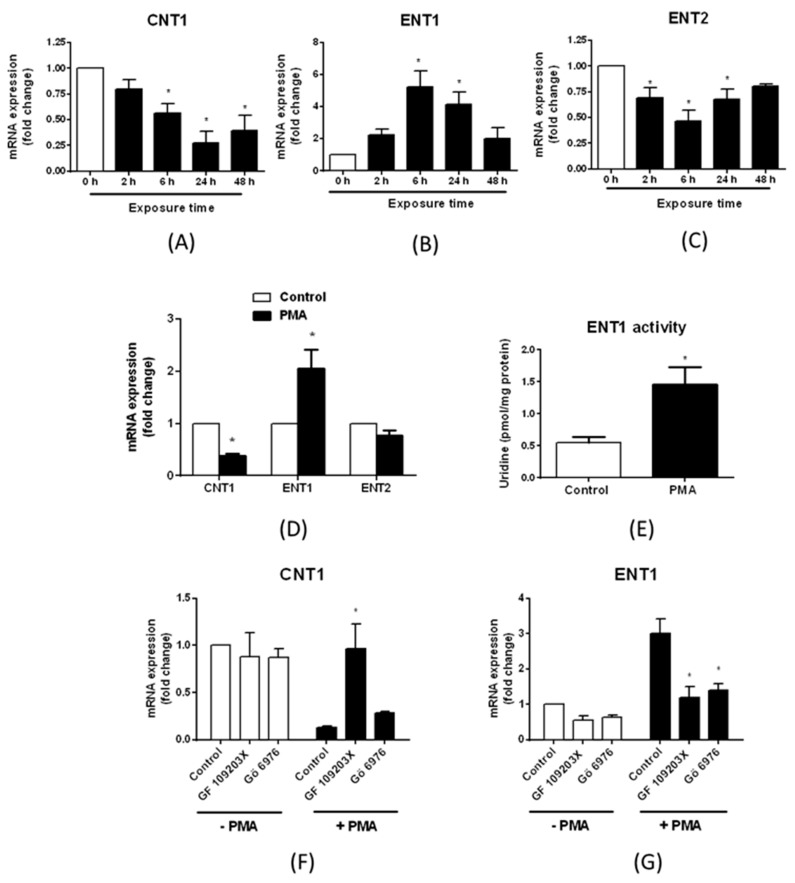
Effects of the protein kinase C (PKC)-activating agent phorbol myristate acetate (PMA) on transporter nucleoside expression in differentiated HepaRG cells. (**A–C**) Differentiated HepaRG cells were exposed to 100 nM PMA for various lengths of time (0 to 48 h). Expression of (**A**) CNT1, (**B**) ENT1 and (**C**) ENT2 mRNAs was next analyzed by RT-qPCR. Data are expressed as fold change comparatively to untreated cells and are the means ± S.E.M. of values from four independent assays. *, *p* < 0.05 when compared to cells not exposed to PMA. (**D**) Primary human hepatocytes were either untreated (control) or exposed to 100 nM PMA. CNT1, ENT1 and ENT2 expression was next analyzed by RT-qPCR. Data are expressed as fold change comparatively to untreated cells and are the means ± S.E.M. of values from at least three independent hepatocyte populations. *, *p* < 0.05 when compared to cells not exposed to PMA. (**E**) HepaRG cells were either untreated or exposed to 100 nM PMA for 24 h. ENT1 activity was next determined as indicated in Materials and Methods. Data are expressed as pmol uridine/mg protein and are the means ± S.E.M. of values from three independent assays. *, *p* < 0.05 when compared to control cells. (**F**,**G**) HepaRG cells were either untreated or exposed to 100 nM PMA for 24 h in the absence or presence of 2 µM GF 109203X or 5 µM Gö 6976. (**F**) CNT1 and (**G**) ENT1 mRNA expression was next determined by RT-qPCR. Data are expressed as fold change comparatively to untreated cells and are the means ± S.E.M. of values from three independent assays. *, *p* < 0.05 when compared to control cells not exposed to PKC inhibitors.

PKC activation regulates expression of various sinusoidal and canalicular drug transporters in human hepatic cells [33]. To determine whether it may also impact nucleoside transporter expression, we exposed HepaRG cells to the PKCs-activating agent PMA for various lengths of times (from 2 h to 48 h) and analyzed CNT1, ENT1 and ENT2 mRNA expression. As shown in Figure 8A–C, treatment by PMA for 6 h and 24 h repressed CNT1 and ENT2 mRNA levels, and concomitantly induced those of ENT1; a shorter exposure time (2 h) also down-regulated ENT2, but did not impact CNT1 or ENT1 mRNA expression, whereas a longer exposure time (48 h) repressed CNT1, without significantly altering expression of ENT1 or ENT2. Treatment of primary human hepatocytes by PMA for 24 h also reduced CNT1 mRNA expression, and induced that of ENT1 (Figure 8D); ENT2 expression was concomitantly slightly decreased, without reaching statistical significance. Treatment of HepaRG cells by PMA for 24 h was next shown to increase ENT1 activity by a 2.7-fold factor (Figure 8E). Finally, CNT1 mRNA repression by PMA was found to be prevented by co-treatment with the pan-PKC inhibitor GF 109203X [34], but not with the PKC inhibitor Gö 6976 (Figure 8F), that targets only conventional PKCs [35]. By contrast, both GF 109203X and Gö 6976 inhibited PMA-mediated ENT1 mRNA up-regulation (Figure 8G).

### 3.4. Regulation of Nucleoside Transporter Expression by Inflammatory and Growth Factors

**Figure 9:**
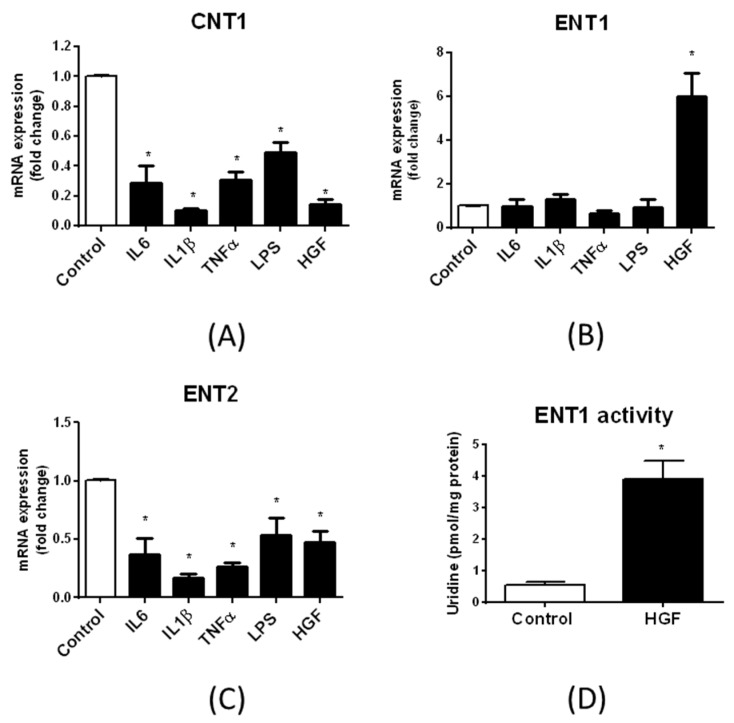

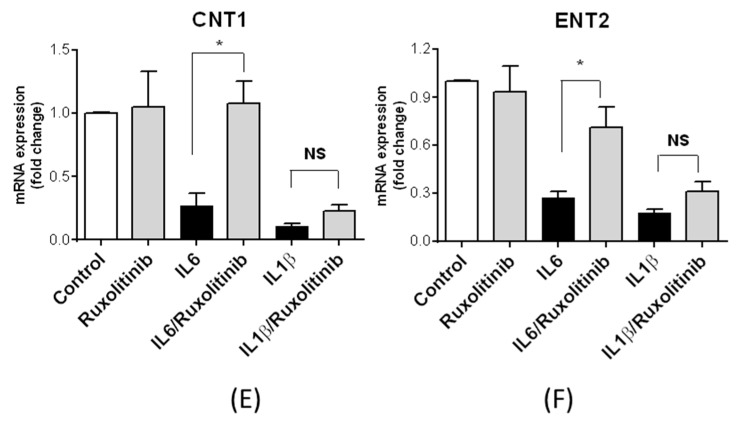
Effects of pro-inflammatory and growth factors on nucleoside transporter expression in differentiated HepaRG cells. (**A**–**C**) Differentiated HepaRG cells were either untreated (control) or exposed to 10 ng/mL IL6, 1 ng/mL IL1β, 10 ng/mL TNFα, 10 ng/mL LPS or 20 ng/mL HGF for 24 h. Expression of (**A**) CNT1, (**B**) ENT1 and (**C**) ENT2 mRNAs was next analyzed by RT-qPCR. Data are expressed as fold change comparatively to untreated control cells and are the means ± S.E.M. of values from at least three independent assays. *, *p* < 0.05 when compared to control cells. (**D**) HepaRG cells were either untreated (control) or exposed to 20 ng/mL HGF for 24 h. ENT1 activity was next determined as indicated in Materials and Methods. Data are expressed as pmol uridine/mg protein and are the means ± S.E.M. of values from three independent assays. *, *p* < 0.05 when compared to control cells. (**E**,**F**) HepaRG cells were either untreated (control), exposed to 5 µM ruxolitinib, 10 ng/mL IL6 or 1 ng/mL IL1β or co-exposed to IL6/ruxolitinib or IL1β/ruxolitinib for 24 h. (**E**) CNT1 and (**F**) ENT2 mRNA expression was next determined by RT-qPCR. Data are expressed as fold change comparatively to untreated control cells and are the means ± S.E.M. of values from three independent assays. *, *p* < 0.05; NS, not significant.

Expression of human hepatic drug transporters is well known to be regulated by inflammatory factors, including the inflammatory cytokines IL6, IL1β and TNFα [24], and by growth factors like HGF [36]. To determine whether these factors may also impact the expression of nucleoside transporters in human hepatic cells, HepaRG cells were exposed to the inflammatory cytokines IL1β, IL6 or TNFα, to the reference inflammatory agent LPS or to HGF for 24 h. ENT1, ENT2 and CNT1 mRNA expression was next determined by RT-qPCR (Figure 9A–C). Inflammatory cytokines, LPS and HGF decreased expression of CNT1 (Figure 9A) and ENT2 (Figure 9C). By contrast, ENT1 mRNA levels were not impaired by IL1β, IL6, TNFα and LPS, whereas they were markedly induced by HGF (by a 6.0-fold factor) (Figure 9B). HGF treatment was next demonstrated to concomitantly induce ENT1 activity (Figure 9D). Finally, the Janus kinase (JAK) inhibitor ruxolitinib, which has previously been shown to reverse IL6-mediated suppression of the hepatic sinusoidal drug transporters sodium-taurocholate cotransporting polypeptide (NTCP/*SLC10A1*), OATP1B1 and OCT1 [37], also hindered IL6-mediated down-regulation of CNT1 (Figure 9E) and ENT2 (Figure 9F). By contrast, it failed to block IL1β-mediated suppression of CNT1 (Figure 9E) and ENT2 (Figure 9F) mRNA expression.

## 4. Discussion

In the present study, the human hepatoma HepaRG cell line was characterized with respect to expression, activity and regulation of nucleoside transporters. HepaRG cells cultured in the presence of DMSO were found to exhibit a nucleoside transporter mRNA profile similar to that found in freshly isolated human hepatocytes or primary human hepatocytes, i.e., they express CNT1, ENT1 and ENT2, but not CNT2 and CNT3. This likely confirms the highly differentiated status of DMSO-exposed HepaRG cells, which display expression of main hepatic detoxifying proteins [38]. By contrast, CNT1, unlike ENT1 and ENT2, was not expressed at the mRNA level in proliferating HepaRG cells, known to exhibit a low level of hepatic differentiation [1]. Such data suggest that CNT1 can be considered as a differentiation marker for human hepatic cells, which fully agrees with its previously published expression profile, i.e., CNT1 expression is restricted to the liver, kidney and small intestine, whereas, ENT1 and ENT2 are more widely distributed [39]. The absence or very low level of CNT2 mRNA expression in HepaRG cells and freshly isolated human hepatocytes agrees with previous studies which detected no substantial transcript expression of this nucleoside transporter in human liver [40,41] and in suspended human hepatocytes [20]. By contrast, CNT2 transcripts have been reported in sandwich-cultured human hepatocytes [19], thus suggesting that this particular three-dimensional mode of culture for human hepatocytes may induce CNT2 expression. CNT2 may alternatively be in fact present in the human liver, but only at a level lower than those found for ENT1, ENT2 and CNT1 [18]. mRNA expression of CNT3, known to be weakly present in the human liver [42], was not detected in both proliferating and differentiated HepaRG cells, freshly isolated human hepatocytes and primary cultured hepatocytes; it was similarly present at only low or trace level in sandwich-cultured human hepatocytes [19] and suspended human hepatocytes [20].

For proliferating HepaRG cells, expression of ENT1 transcripts was associated with detection of both ENT1 protein at the plasma membrane and ENT1 activity, i.e., sodium-independent and 100 nM NBMPR-inhibitable transport of uridine, which represents nearly the totality of uridine uptake in these cells. ENT1 activity in differentiated HepaRG cells was also the main contributor to total uridine uptake. However, it was much lower than that found in proliferating HepaRG cells. ENT1 protein was, moreover, not obviously detected at the plasma membrane of differentiated HepaRG cells, which may be consistent with a low level of ENT1 expression at this plasma membrane. By contrast, ENT1 was clearly detected in the nuclei of differentiated HepaRG cells, as well as those of proliferating counterparts. Such a nuclear location of ENT1 is rather unusual and has not been previously reported in sandwich-cultured human hepatocytes and in the human liver, which rather mainly express ENT1 at the sinusoidal membrane, although intracellular location in endocytosis vesicles has also been reported [19]. The nuclear labeling in HepaRG cells may consequently be hypothesized to correspond to an artefact due to the non-specific binding of the used anti-ENT1 antibody to an unidentified ENT1-like protein in the nucleus. The fact that immunolocalization with the anti-ENT1 antibody resulted in nuclear labeling of HAP1 and HAP1 ENT1^△^ cells (Figure S2A) likely supports this hypothesis. ENT2 was also found to be located in the nuclei of proliferating and differentiated HepaRG cells, suggesting that ENT2 expression at the plasma membrane is probably very weak, which agrees with the low contribution of ENT2 activity to total uridine transport in HepaRG cells. It is noteworthy that nuclear localization of ENT2 has previously been reported in other cell types [43,44] and may be implicated in nuclear uptake of nucleosides [45]. CNT1 protein was detected in differentiated HepaRG cells, but not in proliferating counterparts, which agrees with the mRNA expression profiles of these cells. CNT1 was however not located at the sinusoidal plasma membrane, but mainly at the pericanalicular or canalicular domain of differentiated HepaRG cells, which may explain why differentiated HepaRG cells failed to exhibit obvious CNT activity, investigated as a sodium-dependent uridine uptake activity in the present study. By contrast, primary human hepatocytes displayed CNT1 activity, i.e., sodium-dependent uptake of uridine inhibited by thymidine, but not by inosine. Such a result is consistent with the expression of CNT1 at the sinusoidal pole of cultured hepatocytes, knowing that a fraction of CNT1 is also located at the canalicular pole [19]. Besides immunolocalization assays, Western-blotting experiments may have to be considered in the future for more precisely quantifying nucleoside transporter protein expression in both HepaRG cells and primary human hepatocytes. This may permit us to search for a putative correlation between protein expression and the activities of nucleoside transporters.

PKC activation, well-known to modulate expression of various hepatic drug transporters [23,33], was found to regulate expression of nucleoside transporters in differentiated HepaRG cells and primary cultured human hepatocytes. The effects of PMA however depend on the nature of the nucleoside transporters: The phorbol ester repressed both CNT1 and ENT2 mRNA expression and induced that of ENT1. PMA concomitantly enhanced ENT1 activity in differentiated HepaRG cells, indicating that ENT1 regulation by PKC activation occurs at a functional level. ENT1 up-regulation by PMA was hindered by the pan-PKC inhibitor GF 109203X and by the conventional/classical PKC inhibitor Gö 6976, indicating that ENT1 induction implicates activation of a conventional/classical PKC, most likely the PKC-α isoform, which is the main conventional PKC isoform present in HepaRG cells [23]. By contrast, CNT1 repression by PMA was prevented by GF 109203X, but not by Gö 6976, thus suggesting that it may depend on activation of a novel PKC isoform expressed in HepaRG cells, i.e., PKC-δ, PKC-ε or PKC-η [23]. The implication of atypical PKCs, that lack the tandem repeat of cysteine rich motifs corresponding to the PKC binding domain of phorbol esters like PMA [46], has to be discarded.

Inflammatory cytokines such as IL1β, IL6 and TNFα as well as growth factors like HGF constitute other factors known to regulate drug transporter expression in hepatocytes [24,36,47,48], which likely contributes to the overall repression of hepatic detoxification pathways during inflammation [49]. Cytokines have also been demonstrated to regulate expression of nucleoside transporters in certain cell types. CNT1 is thus up-regulated by IL6 and TNFα in rat hepatocytes [50], whereas interferon (IFN) α repressed ENT1 expression and activity in mouse macrophages [51]. ENT1 expression is also suppressed by IL1β in stromal progenitor cells [52], whereas IFNα induced CNT2 expression and activity in the non-transformed human hepatocyte-derived cell line HHL5 [53]. Our data fully confirm the regulation of nucleoside transporter expression by inflammatory cytokines, through demonstrating that IL1β, IL6 and TNFα, as well as the pro-inflammatory factors LPS, decreased ENT2 and CNT1 mRNA expression in differentiated HepaRG cells. The repressing effects of IL6, unlike those of IL1β, were counteracted by the JAK inhibitor ruxolitinib, which is fully consistent with the fact that IL6, but not IL1β, acts through the JAK/Signal transducer and activator of transcription (STAT) signaling pathway on drug transporter expression [37]. Like inflammatory cytokines, HGF reduced CNT1 and ENT2 mRNA expression in HepaRG cells. HGF also markedly increased ENT1 mRNA expression, which, by contrast, was not altered by inflammatory cytokines. HGF concomitantly induced ENT1 activity, indicating that ENT1 up-regulation by HGF was functionally relevant. HGF appears thus as differentially regulating nucleoside transporter in HepaRG cells; it represses CNT1 and ENT2 and induces ENT1. Whether such differential regulations may occur in vivo in response to HGF and what may be their putative physio-pathological consequences in terms of nucleoside transport in human liver would deserve further studies.

DMSO-exposed HepaRG cells are now considered as relevant surrogates for human hepatocytes for drug metabolism, transport and toxicity studies [3,38]. However, HepaRG cells remain transformed hepatoma cells, which express at low level certain detoxifying proteins present in human hepatocytes, such as the cytochromes P-450 1A2, 2D6 and 2E1 and the drug transporters OATP1B3 (*SLCO1B3*) and bile salt export pump (BSEP/*ABCB11*). The absence of detectable CNT1 activity in differentiated DMSO-exposed HepaRG cells constitutes another difference with primary human hepatocytes. In this context, however, it is noteworthy that ENT1 activity remains the main nucleoside transporter activity contributing to uridine uptake in both differentiated HepaRG cells and primary human hepatocytes. Moreover, ENT1 is considered to be the major contributor to the hepatic uptake of nucleoside analogs such as the antiviral drug ribavirin [20,21]; in this way, ENT1 expression variations may account for differences in response rate in patients receiving ribavirin-based anti-hepatitis C virus therapy [54]. Therefore, such data suggest that the use of differentiated ENT1-expressing HepaRG cells may be relevant for the analysis of cellular pharmacokinetics and metabolism of nucleoside-like drugs, especially of those mainly transported by ENT1. The relevance of HepaRG cells for nucleoside transporters-related studies is also supported by the fact that HepaRG cells exhibit nucleoside transporter regulations in response to various effectors such as PKCs, inflammatory cytokines and the growth factor HGF. 

In summary, HepaRG cells were found to exhibit mRNA expression of the nucleoside transporters ENT1, ENT2 and CNT1, and to display notable ENT1 activity. Nucleoside transporters, especially ENT1, can consequently be added to the list of drug transporters expressed and functional in human hepatoma HepaRG cells. 

## Figures and Tables

**Table 1:** Differential sensitivity of nucleoside transporters to inhibitors.

Inhibitor	Nucleoside Transporter				
	CNT1	CNT2	CNT3	ENT1	ENT2
Sodium whithdrawal	+	+	+	−	−
*S*-(4-Nitrobenzyl)-6-thioinosine (NBMPR) (100 nM)	−	−	−	+	−
NBMPR (100 µM)	−	−	−	+	+
Inosine (200 µM)	−	+	+	Not relevant	Not relevant
Thymidine (200 µM)	+	−	+	Not relevant	Not relevant

+ inhibition; − no inhibition.

## Supplementary Materials

The following are available online at http://www.mdpi.com/1999-4923/10/4/246/s1, Figure S1: 18S rRNA expression in proliferating and differentiated HepaRG cells; Figure S2: Nucleoside transporter mRNA expression and uridine uptake in (A) human hepatoma HuH-7 cells and (B) human macrophages; Figure S3: ENT1 expression and uridine uptake in HAP1 and HAP1 ENT1^△^ cells.
