# Supplementary material for: mRNA Expression and Activity of Nucleoside Transporters in Human Hepatoma HepaRG Cells
Source: Pharmaceutics. 2018 Nov 21;10(4):246. doi: 10.3390/pharmaceutics10040246 (PMC6320972; doi:10.3390/pharmaceutics10040246)
Supplement: Supplementary file 1 [file pharmaceutics-10-00246-s001.pdf]

pharmaceutics

# Supplementary materials: mRNA Expression and Activity of Nucleoside Transporters in Human Hepatoma HepaRG Cells

**Abdullah Mayati, Amélie Moreau, Elodie Jouan, Marie Febvre-James, Claire Denizot, Yannick Parmentier, Olivier Fardel**

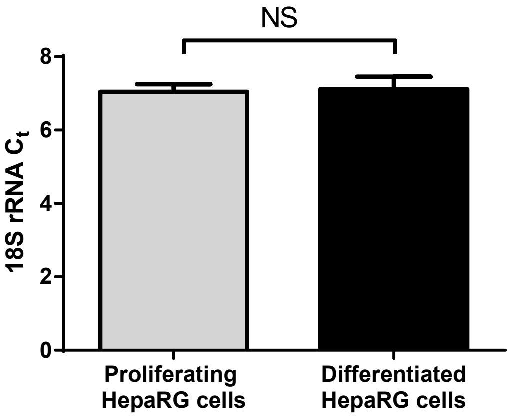


**Figure S1.** 18S rRNA expression in proliferating and differentiated HepaRG cells. Expression of 18S rRNA was determined by RT-qPCR, as described in Materials and Methods. Data are expressed as $C_t$ numbers and are the means ± S.E.M. of 6 assays. NS, not statistically significant ($p > 0.05$, Student's *t*-test).

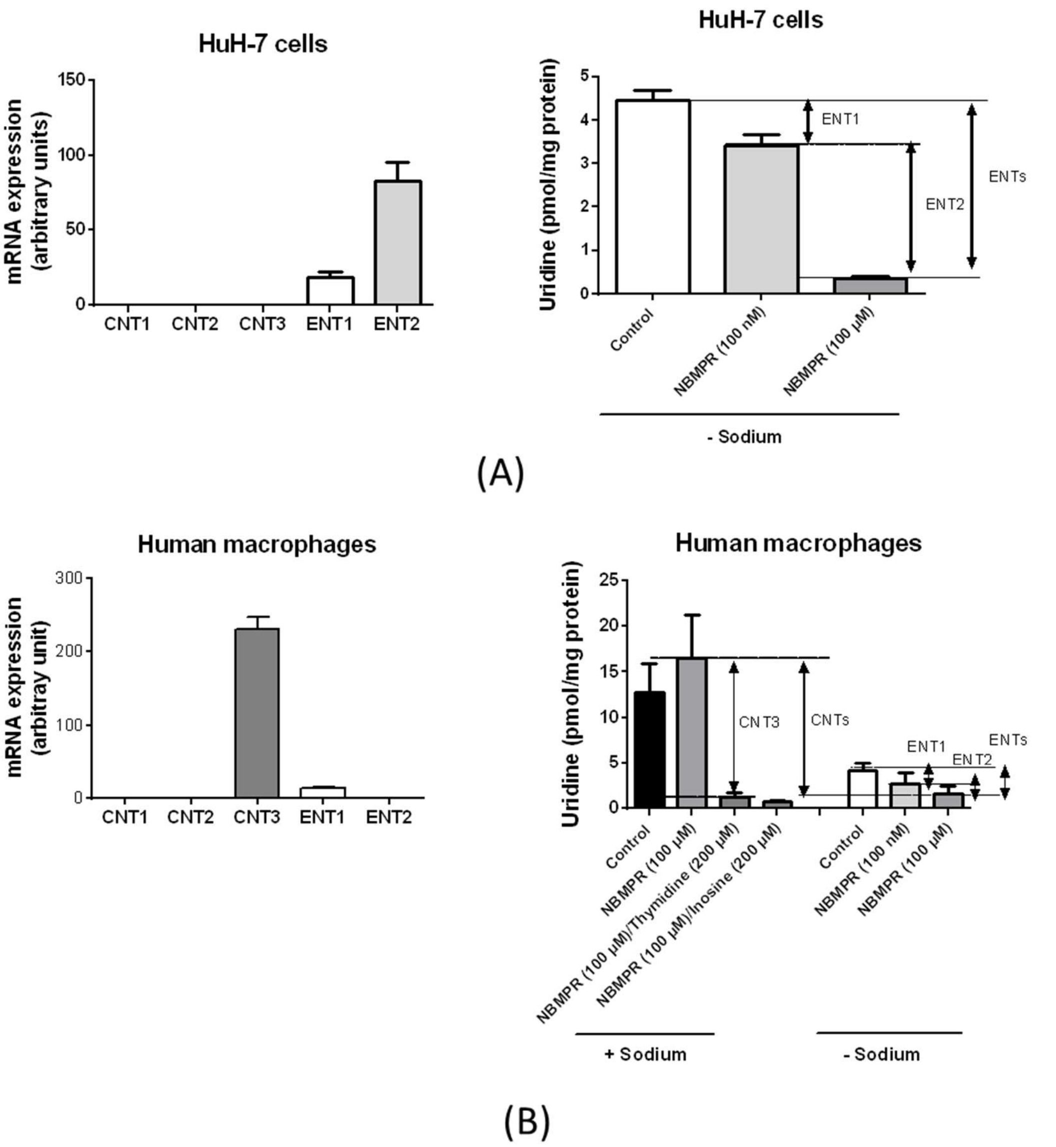


**Figure S2.** Nucleoside transporter mRNA expression and uridine uptake in (**A**) human hepatoma HuH-7 cells and (**B**) human macrophages. (Left panels) Nucleoside transporter mRNA expression was determined by RT-qPCR. Data are expressed in arbitrary units and are the means ± S.E.M. of values from eight independent assays. (Right panels) Cells were incubated with 24.5 nM [$^3$H]-uridine for 5 min at 37°C, in the presence or absence of sodium and in the presence or absence of the ENT inhibitor NBMPR (used at 100 nM or 100 μM). Macrophages were additionally treated by the CNT inhibitors thymidine or inosine. Uridine accumulation was next determined by scintillation counting. Data are expressed as pmol uridine/mg protein and are the means ± S.E.M. of values from three independent assays. Activities of CNTs, CNT3, ENTs, ENT1 and ENT2, defined by equations described in Materials and Methods, are indicated by double arrows on the graphs.

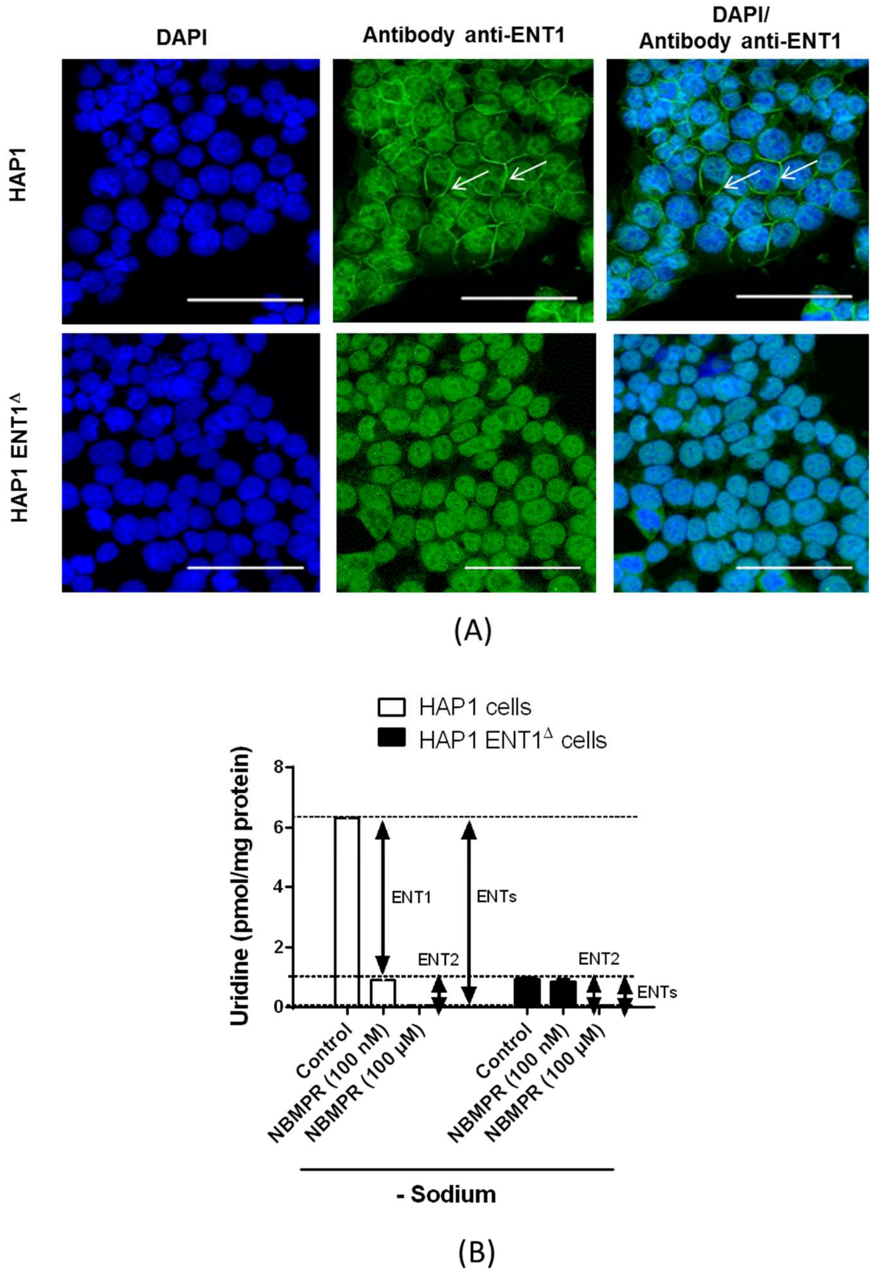


**Figure S3.** ENT1 expression and uridine uptake in HAP1 and HAP1 ENT1$^{\triangle}$ cells. (**A**) ENT1 was immunolocalized, as described in Materials and Methods. ENT1 labelling appears as green fluorescence on fluorescence microscopy pictures, whereas DAPI-stained nuclei are blue. White arrows indicate ENT1-related membrane labelling. Data are representative of three independent assays. White bar = 50 µm. (**B**) Cells were incubated with 24.5 nM [$^{3}$H]-uridine for 5 min at 37°C, in the absence of sodium and in the presence or absence of the ENT inhibitor NBMPR (used at 100 nM or 100 µM). Uridine accumulation was next determined by scintillation counting. Data are expressed as pmol uridine/mg protein and are the means ± S.E.M. of values from three independent assays. Activities of ENTs, ENT1 and ENT2, defined by equations described in Materials and Methods, are indicated by double arrows on the graphs.
